# Multifunctional, Polyurethane-Based Foam Composites Reinforced by a Fabric Structure: Preparation, Mechanical, Acoustic, and EMI Shielding Properties

**DOI:** 10.3390/ma11112085

**Published:** 2018-10-25

**Authors:** Hongyang Wang, Ting-Ting Li, Liwei Wu, Ching-Wen Lou, Jia-Horng Lin

**Affiliations:** 1 Innovation Platform of Intelligent and Energy-Saving Textiles, School of Textiles, Tianjin Polytechnic University, Tianjin 300387, China; jobwang1990@163.com (H.W.); wuliwei@tjpu.edu.cn (L.W.); cwlou@asia.edu.tw (C.-W.L.); 2 Tianjin and Ministry of Education Key Laboratory for Advanced Textile Composite Materials, Tianjin Polytechnic University, Tianjin 300387, China; 3 Department of Bioinformatics and Medical Engineering, Asia University, Taichung 41354, Taiwan; 4 Department of Chemical Engineering and Materials, Ocean College, Minjiang University, Fuzhou 350108, China; 5 College of Textile and Clothing, Qingdao University, Shandong 266071, China; 6 Department of Medical Research, China Medical University Hospital, China Medical University, Taichung 40402, Taiwan; 7 Laboratory of Fiber Application and Manufacturing, Department of Fiber and Composite Materials, Feng Chia University, Taichung 40724, Taiwan; 8 Department of Fashion Design, Asia University, Taichung 41354, Taiwan; 9 School of Chinese Medicine, China Medical University, Taichung 40402, Taiwan

**Keywords:** fabric reinforced composites, sound absorption, compression resistance, electromagnetic interference shielding effectiveness (EMI SE), drop impact

## Abstract

This study proposes multifunctional, fabric-reinforced composites (MFRCs) based on a bionic design, which are prepared by two-step foaming and a combination of different fabric constructs. MFRCs are evaluated in terms of sound absorption, compression resistance, electromagnetic interference shielding effectiveness (EMI SE), and drop impact, thereby examining the effects of fabric structures. The test results indicate that the enhanced composites have superiority functions when combined with carbon fabric in the upper layer and spacer fabric in the lower layer. They have maximum compression resistance, which is 116.9 kPa at a strain of 60%, and their compression strength is increased by 135.9% compared with the control specimen. As a result of the fabric structure on the cell morphology, the maximum resonance peak shifts toward high frequency when using spacer fabric as the intermediate layer. The average sound absorption coefficient is above 0.7 at 1000–4000 Hz. The reinforced composites possessed EMI SE of 50 dB at 2 GHz; an attenuation rate of 99.999% was obtained, suggesting a good practical application value. Furthermore, the cushioning effect of the MFRCs improved significantly, and the maximum dynamic contact force during the impact process was reduced by 57.28% compared with composites without any fabric structure. The resulting MFRCs are expected to be used as sound absorbent security walls, machinery equipment, and packaging for commercial EMI shielding applications in the future.

## 1. Introduction

Materials composed of bio-inspired structures have been gaining in popularity in the academic field in recent years [1,2]. For example, pomelo and coconut biomaterials have an intrinsic porous gradient structure, which is able to dissipate a high-energy impact to protect the flesh [3,4]. Conversely, there are few studies on the acoustic properties of biomaterials. Due to their light weight and high porosity, porous materials received much attention in the twentieth century [5]. Flexible polyurethane foam (FU) has been commonly used in the sound absorption field due to its low cost and ease of processability [6]. Polyurethane foam primarily uses an internal porous structure and restrained air to dissipate the energy of sound waves, thereby obtaining sound absorption efficacy [7,8].

The majority of studies on polyurethane composites focus on the addition of functional particles to improve their sound-absorbing efficiency [9,10] and mechanical properties [11]. Baferani et al. blended multi-walled carbon nanotubes (CNTs) and polyol for a long time using ultrasonic dispersion in order to strengthen the reaction of CNTs’ surface. After free foaming, there was a significant raise in the sound absorption performance of the composites [12]. Sung et al. combined flake-like functional fillers with polyol for foaming in order to examine the influence of the fillers on the sound absorption coefficient. The composites were proven to have a greater sound absorption coefficient in high-frequency regions [13]. Pan et al. investigated the influence of warp-knitted spacer fabrics on spacer fabric/rigid polyurethane foam composites and found that the addition of warp-knitted spacer fabrics had a positive influence on sound absorption [14]. Lin et al. found that the PET/TPU/PU sandwich composite plank made by the laminated method successfully achieved the sound absorption coefficient of above 0.9 at high frequencies [15]. Asadi Khanouki et al. investigated the effect of SiO_2_ nanoparticles on the acoustic damping based on the content, nanometer sizes, and cell distribution, which provided the reference of high-sound-absorbing materials [16]. Recently, Jiang et al. developed a layered composite consisted of alternating layers of PU foam and BT/NBR composite, which has an excellent sound absorption property at low frequency [17]. In summary, multiple mechanisms and structures should be considered in designing sound-absorbing materials.

In addition to the sound absorption of polyurethane foams, the improvement of the electromagnetic shielding property of polyurethane foams also has been a popular research topic [18]. Most scholars use pellets or fibers with electric conduction to obtain high electromagnetic shielding effectiveness (EMI SE) [19]. Jeddi et al. added conductive fillers to improve the EMI SE of the foams [20]. Carbon fibers have excellent electrical properties, so that using a general wet-laid method can produce the carbon fabric with high EMI SE [21]. Similarly, Ameli et al. studied the effects of functional pellets and carbon fibers on the foaming process, and the resulting composites had an EMI SE of 20 dB [22]. Nevertheless, studies on the sound absorption and compression resistance of multifunctional, fabric-reinforced composites (MFRCs) are relatively rare.

The dynamic impact resistance response is another important function of porous foam materials. Exclusively using a foam or fabric structure for cushioning does not yield strong results over all mechanical features, which limits their applications. Hence, a combination of both materials is thus commonly combined in the previous study [23]. Huang et al. proposed flexible sandwich composites using fabrics as cover sheets to enclose a PU-foam-embedded spacer fabric. The composites were strengthened from the interior and had bursting resistance and low velocity impact resistance [24]. Some scholars studied composites that were composed of six varieties of structural parameters, examining their influences on the impact properties of the composites, in an attempt to improve the buffer effectiveness without damaging the spacer fabric inter layer [25]. These studies only analyzed the interaction mechanism between fabric and PU foam and did not focus on the reinforcement and mechanism of the fabric type on the composites. Therefore, this study proposes producing MFRC using a two-step foaming technique. Polyurethane foams with different cell structures serve as the matrices, while fabrics that are used as reinforcing interfaces and carbon fabrics are also used for EMI SE. The MFRCs are evaluated in terms of compression resistance, sound absorption, EMI SE, and impact resistance in order to examine the effect of fabric structure and the interfacial reaction mechanism. The findings can serve as a reference for the development of composites with multiple functions and high performance.

## 2. Experimental

### 2.1. Materials

Flexible polyurethane foams are made of polyether polyol with a specific weight of 1.05 g/cm^3^ (CST-1076A Keshengda Trading, Shenzhen, China) and Isocyanate with a specific weight of 1.25 g/cm^3^ (CST-1076B, Keshengda Trading, Shenzhen, China). The carbon fabrics (Chin Carbon Fiber Technology, Yixing, China) have a warp density of 6 ends/inch, a weft density of 6 picks/inch, a carbon fiber fineness of 12 K, a basis weight of 390 g/m^2^, and tensile load of 1020 N. Nylon nonwoven fabrics (Far Eastern New Century Corporation, Taipei, Taiwan) have an areal density of 200 g/m^2^. The warp-knitted spacer fabrics (YT-0638, Huayu Weaving, Jinjiang, Fujian, China) have a mesh size of 4 mm, a thickness of 5 mm, top/bottom layers being composed of 200 D polyester monofilaments, and a spacer layer being composed of 30 D polyester monofilaments.

### 2.2. Preparation of Free-Foaming Polyurethane Gradient Composites

Flexible polyurethane foams (FU) with different cell structures are made using the one-step foaming process. At room temperature, deionized water (ratios of water to polyol are 0, 0.5, and 1 wt %) and polyether polyol are blended at 800 rpm for five minutes, after which isocyanate is added and blended at 1200 rpm for another ten seconds. The mixtures are infused into a mold for free foaming and curing at room temperature (25 °C) and normal atmospheric pressure. Samples are denoted as FU0, FU0.5, and FU1, in which “FU” stands for flexible polyurethane foams, and the digit stands for the content of deionized water.

**Figure 1:**
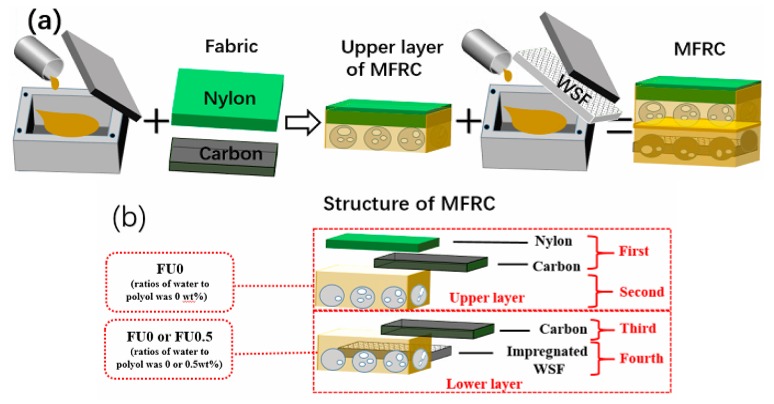
(**a**) Manufacturing process and (**b**) structural diagram of MFRCs.

Multifunctional, fabric-reinforced composites (MFRCs) are made using the two-step foaming process. Polyol and isocyanate are blended at 1200 rpm for ten seconds. The mixture is infused into a mold (300 mm × 300 mm × 10 mm) quickly and then covered with a selected fabric before the mold is sealed, forming the upper layer of MFRCs. Next, deionized water and polyol with an optimal ratio are blended, after which isocyanate is added and blended at 1200 rpm for another ten seconds. The mixtures are infused into another mold (300 mm × 300 mm × 20 mm) and covered with the upper layer for free foaming and curing, forming the MFRCs as seen in Figure 1.

The manufacture of fabric-reinforcing MFRCs involves the upper and lower layers. A nylon fabric or/and a carbon fabric are used to cover the deionized, water-free foaming mixture in the mold for free foaming and curing, forming the upper layer. Then, the foaming mixtures containing 0 wt % or 0.5 wt % deionized water saturate a warp-knitted spacer fabric (WSF) to form the lower layer. Table 1 shows the denotations and variations of interface-reinforcing MFRCs.

### 2.3. Characterizations

**Figure 2:**
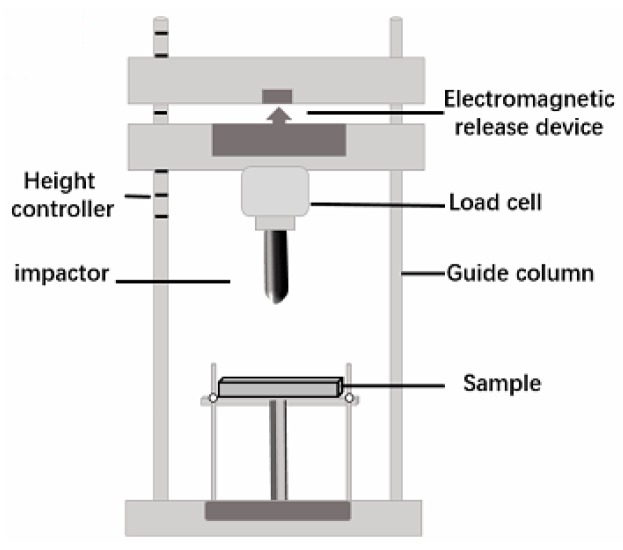
Drop-weight impact tester.

The microstructure (i.e., cavities and interconnected pores) of polyurethane foam is observed with a scanning electron microscope (SEM, TM-1000, Hitachi, Tokyo, Japan) at 30 kV. The SEM images are used to analyze the cell properties (i.e., the thickness of strut, the cavity, and pore sizes). The density of samples is measured as specified in ASTM D3574-17 [26]. Samples have a size of 50 mm × 50 mm × 10 mm. The volume of each sample is measured at five random spots using a vernier caliper, while the mass of samples is weighed using an electronic balance (Shanghai Pu Chun Measure Instrument, Shanghai, China), thereby computing the density. The Fourier transformed infrared spectroscopy (FTIR Frontier, Nicolet, Thermo Fisher Scientific, Waltham, USA) with an attenuated total reflectance (ATR) accessory under unforced condition is used for the functional group analysis. The FTIR spectra are obtained from 32 scans with a resolution of 4 cm^−1^. The compressive strength of samples is measured at a rate of 5 mm/min using a universal testing machine (HT-2402, Hong Ta Instrument Co., Ltd., Taipei, Taiwan), as specified in ASTM D 3574-17 [26]. Samples have a size of 50 mm × 50 mm × 20 mm. As specified in the ASTM E1050-12 [27], a twin-microphone impedance tube (Automotive Research & Testing Center, Taichung, Taiwan) is used to measure the sound absorption of samples at a frequency of 100–4000 Hz based on the transfer function method. The samples have a cylindrical shape with a diameter of 38 mm. The air chamber size is 10 mm lengthwise. EMI shielding performance of the composite fabrics is evaluated using an Agilent E5063A vector network analyzer (US), as specified in ASTM D4935-18 [28]. All samples are sliced into circular plates with a diameter of 100 mm. The power coefficients of reflectivity (R) and the absorptivity (A) could be acquired according to the measured scattering parameters (S11 and S21). The surface resistance of the material is tested using a multimeter (Victor Hi-tech Co., Ltd., Shenzhen, China). A stereomicroscope (SMZ-10A, Nikon, Tokyo, Japan) is used to observe the morphology of the fractured surface of samples in order to analyze the structure. The drop-weight impact tester is manufactured by Xin Zhi Electronic Automation Company in Taichung, Taiwan. As shown in Figure 2, the impactor is released from a specified height to impact the sample (100 mm × 100 mm) mounted on the anvil. The impactor made of polished steel weighs eight kilograms and has a bullet-shape with a 10-mm diameter. The impact contact force applied to the specimen is measured by load cell that is fitted on top of impactor, as specified in ASTM D1596-14 [29]. In the experiment, the impact energy is 10 J, and the surface with nylon fabric is selected as the impact surface. Five samples for each specification are used for the tests.

## 3. Results and Discussion

### 3.1. Effects of Water Content on Cell Structure

**Figure 3:**
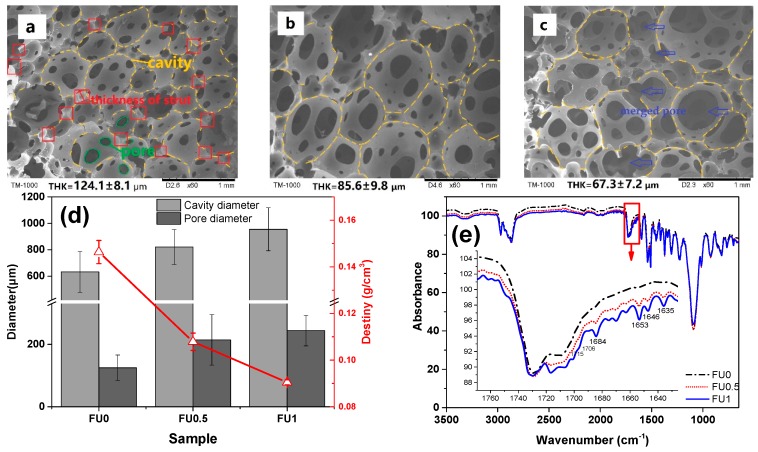
Effects of water content on polyurethane foam. Microscopic images of FU as related to the content of deionized water being (**a**) 0, (**b**) 0.5, and (**c**) 1 wt %. (**d**) is the cavity, pore diameter, and volume density, and (**e**) is the FTIR spectrum of the samples.

Figure 3 shows the microstructure of FU series that is made using the one-step foaming. The diameter of cavities, the diameter of interconnected pores, and the thickness of struts demonstrate significant variations in the cell structure of FU series. The thickness of the strut is 124.1 μm for FU0 (Figure 3a) and 85.6 μm for FU0.5 (Figure 3b). The variation in the thickness of the strut becomes mild for FU1 (Figure 3c). The cavities are irregularly formed, and there are also merged pores. Being highly dependent on the cell structure, the volume density has a great influence on the sound absorption and mechanical properties of FU series [9]. Figure 3d shows that FU0 has the highest bulk density, which then decreases as a result of increasing the deionized water. The observed trend is basically in conformity with that of the thickness of strut.

Regardless of whether it is the cavities or the pores, the average diameter increases when there is more deionized water added, but the opposite is the case for the thickness of strut. The presence of water molecules accelerates the foaming reaction, which generates more CO_2_ that expedites the growth of cavities and enlarges the interconnected pores [30]. However, FU1 has a steadily slow decrease in the number of cavities and pores. Within a specified space, large cavities limit the growth and the size of other cavities. The thickness of strut becomes small, and FU1 thus has a low bulk density [31]. Figure 3e shows the FTIR spectra of FU0, FU0.5, and FU1 as related to the content of the deionized water. There are no significant variations in characteristic peak of polyurethane, which indicates that deionized water does not interfere with the chemical reaction of foaming, and the three exceptions are characteristic peaks at 1750–1620 cm^−1^ corresponding to the presence of carbonyl group at 1730, 1715, and 1706 cm^−1^. The characteristic peaks at 1715 cm^−1^ and 1706 cm^−1^ are intensified, while the characteristic peak 1730 cm^−1^ remains the same when the deionized water increases. Urea carbonyl group has corresponding characteristic peaks occurring at 1684, 1670, 1653, 1646, and 1635 cm^−1^ [32]. The characteristic peaks are intensified as a result of increasing deionized water, because more deionized water helps increase the amount of CO_2_ as well as urea groups. Moreover, the urea carbonyl group has a more significant intensification based on the spectrum. Meanwhile, some free carbonyl at 1684 cm^−1^ directly react with -NCO to form urea carbonyl group that consists of hydrogen bond at 1653, 1646, and 1635 cm^−1^. Therefore, more deionized water results in more CO_2_ and greater transformation of urea. The corresponding urea carbonyl group fluctuates to a greater extent and has a higher reaction rate, which in turn causes the presence of hydrogenated carbamate carbonyl group at 1715 cm^−1^.

The content of deionized water is a crucial factor to the diameters of cavities and interconnected pores. In particular, 1 wt % of deionized water expedites the foaming process, which triggers the rapid formation of cavities and pores as well as the merged pores (Figure 3c). The thickness of strut decreases and the FU series has a low mechanical property and a poor foaming quality. Hence, FU0 and FU0.5 are used for the matrices to be combined with fabrics to reinforce the structure of MFRCs.

### 3.2. Effects of Structure on Compression Resistance of MFRCs

**Figure 4:**
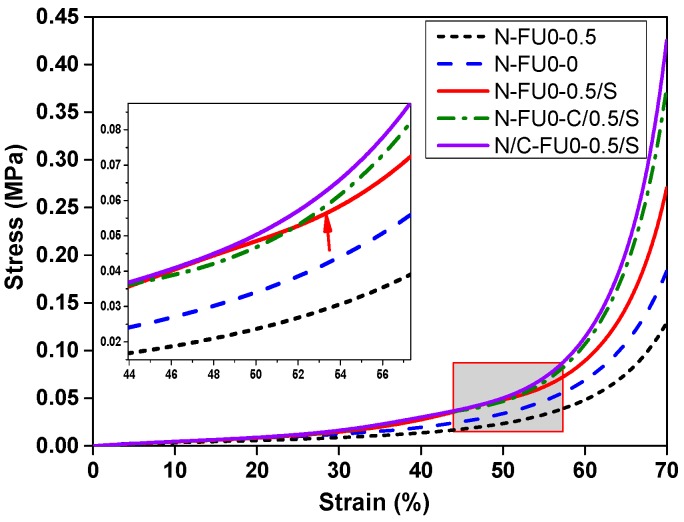
Stress-strain curves of MFRCs.

Figure 4 shows the compression resistance of MFRCs, which are in conformity of that of fruit wall of Citrus maxima at a strain of 40% in a previous study [3]. In a plateau region, composites with spacer fabric have similar stress resistance. However, N/C-FU0-0.5/S has the greatest compressive capacity during the initial densification stage, followed by N-FU0-C/0.5/S, N-FU0-0.5/S, N-FU0-0, and N-FU0-0.5, because the spacer fabric reinforces the stress tolerance of the bottom layer of MFRCs [25] and polyurethane foam bonds the carbon fabric and nylon fabric, which in turn enhances the compressive strength of MFRCs at the densification stage remarkably. N/C-FU0-0.5/S has the maximum compression resistance, which is 116.9 kPa at a strain of 60%, but the compression resistance of N-FU0-0.5/S and N-FU0-0.5 are only 90.1, 49.5 kPa, respectively. Therefore, the compression strength of the composites with spacer fabric and carbon increase by 135.9%. Additionally, when a carbon fabric is in the middle of N-FU0-C/0.5/S, the stress-strain is lower than that of N/C-FU0-0.5/S in the densification region. When reaching the densification region, polyurethane foam surrounding the intermediate interface compresses the voids between carbon fibers, slowly decreasing the compression stress at a strain of 60%, as seen in the figure.

### 3.3. Effects of Structure on Acoustic Property of MFRCs

**Figure 5:**
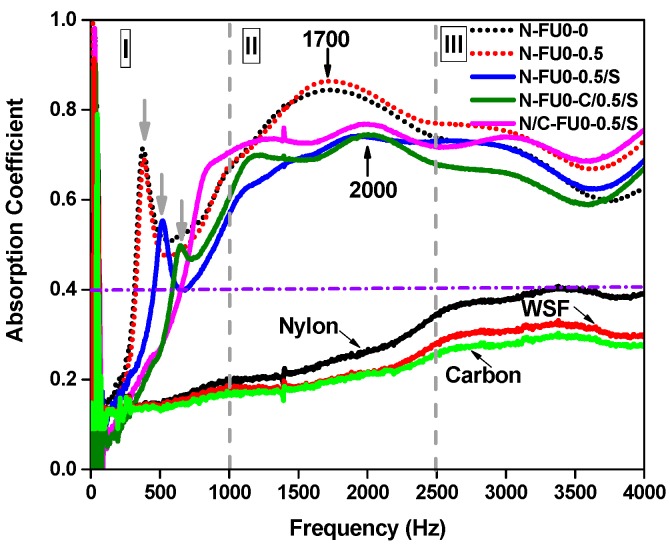
Sound absorption coefficient of MFRCs.

**Figure 6:**
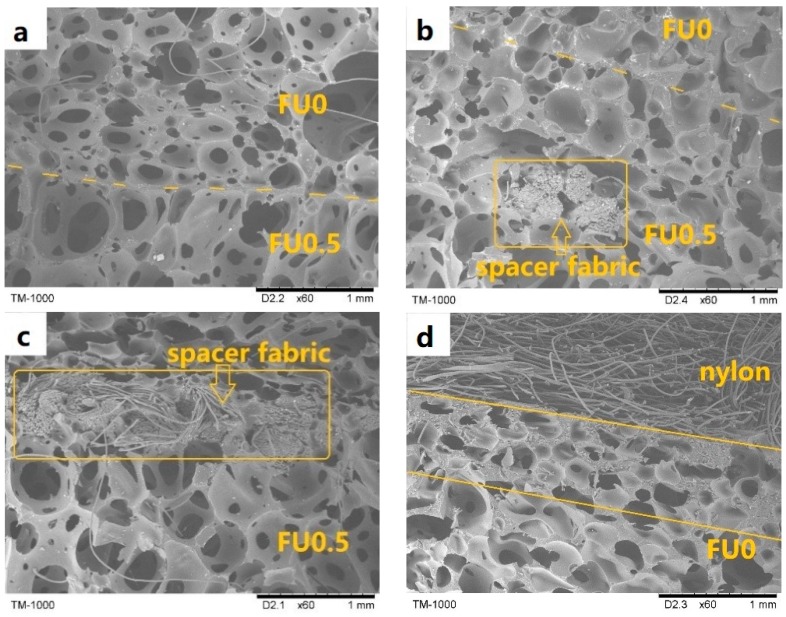

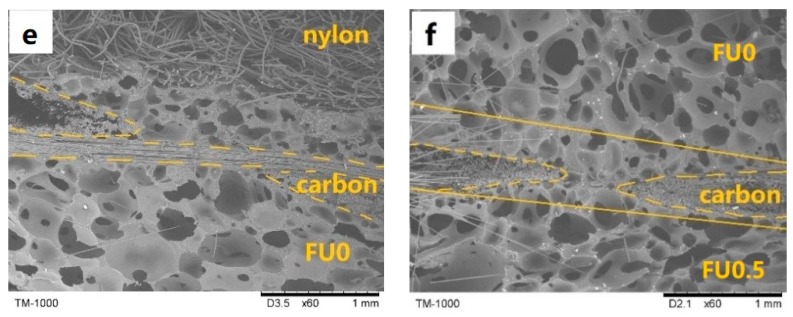
SEM images of fabric and cross section of MFRCs. (**a**) Cross section of interface of upper and lower layer of N-FU0-0.5, (**b**,**c**) cross section of spacing fabric structure and foam, (**d**) cross section of nylon and foam, (**e**) cross section of nylon and carbon fabric of N/C-FU0-0.5/S, and (**f**) cross section of carbon fabric and foam of N-FU0-C/0.5/S.

Porous mediums are divided into fluid and solid phases, and a combination of the two distinct phases is able to attenuate the energy of acoustic waves [16]. Figure 5 shows that the acoustic wave absorption capacity of the fabric structure is considerably low, and the sound absorption coefficient is all lower than 0.4 regardless of the sample type. However, N-FU0-0.5 has the optimal sound absorption efficacy at 1700 Hz and obtains a sound absorption coefficient of 0.85 that is higher than that of N-FU0-0. This result is ascribed to an increase in the cell diameter of the PU foam (Figure 6a). As a result, sound waves enter the cells and interact with air to strengthen the thermal viscosity, thereby improving the sound absorption [33]. Moreover, adding a spacer fabric decreases the sound absorption coefficient of N-FU0-0.5/S, and the maximum sound absorption coefficient thus occurs at a frequency of 2000 Hz. The addition of a spacer fabric affects the nucleus growth of PU foam, resulting in irregular cells between fibers and PU foam, as well as a low porosity (Figure 6b,c). Hence, sound waves have an intensified tortuosity, which affects their transmission path and decreases the sound absorption coefficient of the composites [34].

When carbon fabrics are incorporated, the sound absorption coefficient of the composites is dependent on the position of the carbon fabrics. When the carbon fabrics are in the proximity to the nylon fabrics, N/C-FU0-0.5/S have higher sound absorption than N-FU0-0.5/S with an average sound-absorbing coefficient of above 0.7 at 1000–4000 Hz. Without carbon fabrics, the majority of nylon fabrics are immersed in PU foam, forming a rigid interface (Figure 6d) and adversely affecting the porosity significantly. The combination of carbon fabrics improves the morphology of cells (Figure 6e), which in turn enhances vibration friction efficacy during the transmission of sound waves. In contrast, when carbon fabrics are laminated in the medium of the composites, PU foam permeates them, which affects the cell morphology in the foam–foam interface (Figure 6f). Therefore, the sound absorption coefficient of the sample at high frequencies decreases. In light of the overall structure, the incorporation of fabric structure with the composites changes the cell morphology in the interfaces, and the resonance peak at 0–1000 Hz then shifts toward high frequencies, which is indicated by grey arrows.

### 3.4. Effects of Structure and Interface on EMI SE of MFRCs

**Figure 7:**
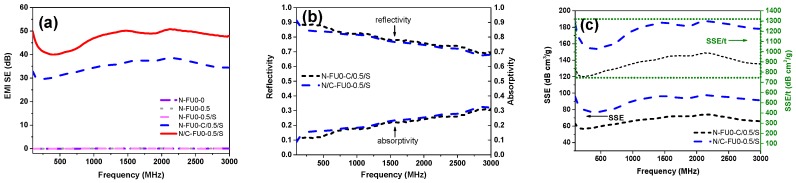
(**a**) EMI SE curves of MFRCs, (**b**) reflectivity and absorptivity of MFRCs, and (**c**) specific SE (SEE) and absolute EMI SE values (SSE/t) of MFRCs.

Figure 7 shows the EMI SE curves of different MFRCs. Without the addition of carbon fabric, MFRCs have almost no EMI SE. N/C-FU0-0.5/S has EMI SE of 40–50 dB, which satisfies the requirement for the commercial EMI shielding applications [35]. Specifically, the EMI SE at 2.0 GHz is 50 dB, indicating only 0.001% transmission through the fabric composite, i.e., 99.999% attenuation of the incident EM radiation calculated according to the formula in the literature [36,37]. It is higher than carbon nanofiber-reinforced syntactic foam with EMI shielding effectiveness of 25 dB [38] and non-woven carbon fabric with EMI shielding effectiveness of 24–28 dB in previous research [39]. The EMI mechanisms can depend on the compact plain structure and intrinsic electric conductivity, which are able to reflect the majority of the electromagnetic waves and attenuate the energy of electromagnetic waves [40]. To clarify the shielding mechanism, the coefficients of reflectivity (R) and the absorptivity (A) are calculated from the measured scattering parameters. As shown in Figure 7b, the R for N/C-FU0-0.5/S and N-FU0-C/0.5/S are more than 70%, indicating the primary shielding mechanism is reflection rather than absorption. This is different from other studies in which composites filled with carbon fiber [22] or graphene oxide [41] show the dominated shielding mechanism of absorption. However, as the frequency increases, the absorption of electromagnetic waves increases. Figure 7c shows the specific SE (SSE) (defined as the SE per unit volume) and absolute EMI SE values (SSE/t) of the composites with carbon fabric. When carbon fabrics are added in the first layer, the SSE and SSE/t of N/C-FU0-0.5/S were enhanced effectively. For example, composite reaches as high as 97 dB·cm^3^·g^−1^ and 1280 dB·cm^3^·g^−1^ at 2 GHz, respectively. Additionally, the beyond and beneath interfaces are photographed using a stereomicroscope to analyze the change of electrical properties.

**Figure 8:**
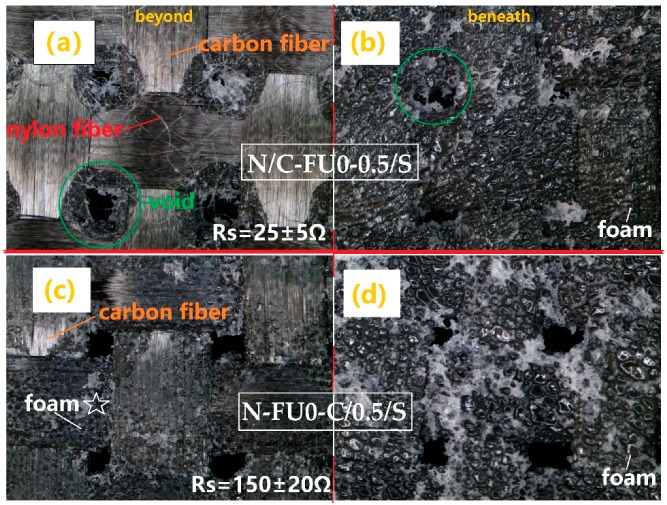
Microscopic images of interface beyond and beneath the carbon fabric. (**a**) Beyond the carbon fabric of N/C-FU0-0.5/S, (**b**) beneath the carbon fabric of N/C-FU0-0.5/S, (**c**) beyond the carbon fabric of N-FU0-C/0.5/S, and (**d**) beneath the carbon fabric of N-FU0-C/0.5/S.

As can be seen from Figure 8, the most significant difference in the interface is that carbon fabric of N-FU0-C/0.5/S is covered by considerable polyurethane foam (see pentastar in Figure 8c) beyond the carbon fabric, but N/C-FU0-0.5/S has a neat and smooth surface (Figure 8a). In the free-foaming process, polyurethane foam expands through the voids in carbon fabric, and the polyurethane fluid bonds the nylon surface, so N/C-FU0-0.5/S shows the nylon fibers clearly. Polyurethane foam that covers the surfaces of carbon fiber is detrimental to the surface resistance (Rs) of carbon fabric due to the insulation of the foam. The surface resistance of carbon fabric increases from 25 ± 5 Ω to 150 ± 20 Ω, and the surface uniformity of the fabric is destroyed, resulting in a decline in the electrical conductivity of the material [22]. As a result, the covered foam in the plain carbon fabric surface debilitates the shielding against electromagnetic waves.

### 3.5. Effects of Structure on Drop Impact Test of MFRCs

**Figure 9:**
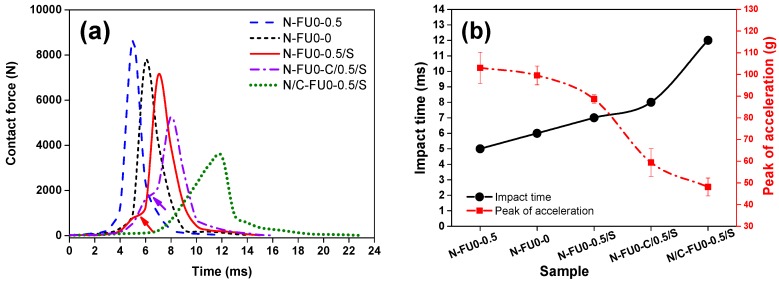
Impact signals at 10 J of MFRCs (**a**) contact force-time curve of MFRCs and (**b**) impact time and peak acceleration of MFRCs.

Figure 9a shows that there are three stages of the time-contact force curves of different samples. Stage I: The contact force gradually increases with the time, which is similar to the plateau stage of static compressive curves. Stage II: The contact force rapidly increases with the time, which corresponds to the densification stage of the static compressive curves of composites. Stage III: The contact force rapidly decreases with the time. Comparing N-FU0-0.5 and N-FU0-0, bulk density of foams is the greatest influence on both static compression and dynamic impact behaviors [42,43]. N/C-FU0-0.5/S has the longest plateau stage and deforms slowly when being impacted, while N-FU0-0.5 has the shortest plateau stage and deforms easily. The more abrupt transition in the initial densification stage is indicated by arrows, which is primarily due to the low impact resistance of the spacer fabric interfaces.

During the impact process, the impact force follows the least resistance path theory. For N-FU0-0.5/S, the compressive resistance of spacer fabric interlayer is higher than that of nylon fabric. The upper foam layer is compressed easily, and an abrupt transition occurs at the initial densification of the spacer fabrics. When carbon fabrics are added in the medium, the medium fabric strength of N-FU0-C/0.5/S increases continuously, and the transitional deformation mitigates, which attenuates the impact force. In contrast, carbon fabrics that are in proximity to the nylon cover sheets can improve the strength and stiffness of the cover sheets significantly, which causes the absence of transition in the curve. Moreover, impact time refers to the required time for the initial value increases to the maximum one. Figure 9b shows the results of computing the peak of contact force and impact time. N/C-FU0-0.5/S has the longest impact time and the lowest corresponding value has the longest plateau stage and deforms slowly. In order to study the effect of fabric structure on the impact process, the fractured surfaces of samples are then observed using a stereomicroscope.

**Figure 10:**
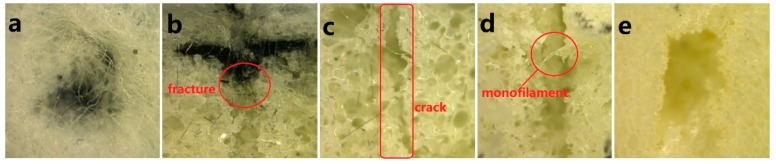
Microscopic images of breaking section of N/C-FU0-0.5/S. (**a**) impact fracture of surface nonwoven fabric; (**b**) cross section of the upper interface of carbon fabric and foam; (**c**) cross section of upper layer of foam; (**d**) cross section of lower layer of spacer fabric; (**e**) impact fracture of lower layer of foam.

In general, the specimens can fracture under horizontal and vertical loads. Hence, crack location and propagation directions are evaluated to determine the type of load that causes specimen failure [44]. Figure 10 shows the cracks that are caused by a vertical load propagate vertically, which is consistent with the findings of the previous study [45]. Figure 10b shows the rupture and breakage of carbon fabrics. In Figure 10c–e, the cracks are not clearly accompanied by broken carbon fibers. When the impact occurs, carbon fabrics start compressing and deforming. The maximum deformation eventually occurs to collide when the anvil, resulting in the breakage of carbon fabrics. This result also proves that the absence of transition in initial densification stage of N/C-FU0-0.5/S in Figure 9a due to the high stiffness and strength of carbon fabrics. According to the theorem of momentum and newton second law, the contact force is in proportion with acceleration. With the same energy, a small acceleration corresponds to a long impact time and a low impact force, which allows the composites to dissipate kinetic energy efficiently and as such obtains greater protection [46]. Comparing to N-FU0-0.5, N-FU0-0.5/S and N/C-FU0-0.5/S have 10.08% and 57.28% lower contact force, respectively. To sum up, N/C-FU0-0.5/S has significant cushioning effect and a higher application value in the engineering field.

## 4. Conclusions

MFRCs are prepared by the adjusting, foam-based structure via 0 and 0.5 wt % of deionized water and using a nylon fabric as the reinforcing surface. The composites have superiority functions when combined with carbon fabric in the upper layer and spacer fabric in the lower layer. N/C-FU0-0.5/S has the highest compression resistance, which is 116.9 kPa at a strain of 60%, indicating that the compressive strength of the composites with spacer fabric and carbon is increased by 135.9% compared with N-FU0-0.5. Furthermore, N-FU0-0.5 has an optimal sound absorption coefficient of 0.86 at 1700 Hz. In particular, the average sound-absorbing coefficient of N/C-FU0-0.5/S is above 0.7 at 1000–4000 Hz, which meets the requirements perfectly. Different locations of a carbon fabric also lead to different EMI SEs, and the EMI SE of N/C-FU0-0.5/S reaches a range between −40 and −50 dB. Specifically, N/C-FU0-0.5/S outperforms N-FU0-0.5 and N-FU0-0.5/S in terms of a low contact force, which suggests a significantly improved buffering efficacy and high value in the practical engineering application.

## Figures and Tables

**Table 1:** Formulation details of MFRCs.

Sample Code	Upper Layer		Lower Layer	
	First	Second	Third	Fourth
N-FU0-0	Nylon	FU0	-	FU0
N-FU0-0.5	Nylon	FU0	-	FU0.5
N-FU0-0.5/S	Nylon	FU0	-	FU0.5/WSF
N-FU0-C/0.5/S	Nylon	FU0	Carbon	FU0.5/WSF
N/C-FU0-0.5/S	Nylon/Carbon	FU0	-	FU0.5/WSF
